# Association of changes in health-related quality of life in coronary heart disease with coronary procedures and sociodemographic characteristics

**DOI:** 10.1186/1477-7525-2-56

**Published:** 2004-10-04

**Authors:** Marijke Veenstra, Kjell I Pettersen, Arnfinn Rollag, Knut Stavem

**Affiliations:** 1 Norwegian Health Services Research Centre; Quality Evaluation Department, P.O. Box 7004, St. Olavs plass 0130, Oslo, Norway; 2 Department of Medicine, Akershus University Hospital, Nordbyhagen, Norway

## Abstract

**Background:**

Few studies have focused on the association between the sociodemographic characteristics of a patient with the change in health-related quality of life (HRQOL) following invasive coronary procedures, and the results remain inconclusive. The objective of the present study was to measure the temporal changes in HRQOL of patients with coronary heart disease, and assess how these changes are associated with invasive coronary procedures and sociodemographic characteristics.

**Methods:**

This was a prospective study of 254 patients with angina pectoris and 90 patients with acute coronary syndrome. HRQOL was assessed with the multi-item scales and summary components of the SF-36, both 6 weeks and 2 years after baseline hospitalization in 1998. Paired *t*-tests and multiple regression analyses were used to assess temporal changes in HRQOL and to identify the associated factors.

**Results:**

Physical components of HRQOL had improved most during the 2 years following invasive coronary procedures. Our findings indicated that patients with angina pectoris who were younger, male, and more educated were most likely to increase their HRQOL following invasive coronary procedures. When adjusting for baseline HRQOL scores, invasive coronary procedures and sociodemographic characteristics did not explain temporal changes in patients with acute coronary syndrome, possibly due to higher comorbidity.

**Conclusion:**

Sociodemographic characteristics should be taken into account when comparing and interpreting changes in HRQOL scores in patients with and without invasive coronary procedures.

## Background

In the medical treatment of patients with coronary heart disease (CHD), invasive coronary procedures – such as percutaneous coronary intervention – are effective in reducing mortality and morbidity [1,2]. An important supplementary outcome of medical interventions and the processes of health care is health-related quality of life (HRQOL) [3,4]. There is a strong interest in differences in the care and outcomes between sociodemographic groups to optimize population health. Differential temporal changes in HRQOL between diverse sociodemographic groups may be of interest in secondary prevention programs to maximize the benefit from treatment for CHD.

Most studies investigating the association between sociodemographic characteristics and HRQOL in patients with CHD focus on cross-sectional group comparisons [5-11]. Longitudinal studies on the association between sociodemographic characteristics and HRQOL have indicated that being female [12-14] and lower socioeconomic status [15] are associated with less temporal improvement in HRQOL. These studies, however, lacked information on medical interventions and focused on short-term changes lasting only up to 1 year. Studies assessing the effect of invasive coronary procedures on HRQOL, have shown that HRQOL improves after intervention [16-20] to levels similar to population norms [21-23]. Some of these studies were clinical trials and involved very selective populations.

Only few studies have focused on the association of sociodemographic variables with temporal changes in HRQOL following invasive coronary procedures. A recent observational study indicated that higher income is associated with greater improvement in physical HRQOL following invasive coronary procedures [24]. Improvements in physical HRQOL appear to be unrelated to the age of patients [25], whereas elderly patients exhibit a stronger improvement in mental HRQOL after medical intervention [24,26]. However, the association of sex and educational attainment with changes in HRQOL following invasive coronary procedures remains inconclusive.

In the present study of patients with CHD, we aimed to (i) describe the effect of invasive coronary procedures on different domains of HRQOL at both 6 weeks and 2 years after baseline hospitalization, (ii) assess the association between sociodemographic characteristics and temporal changes in HRQOL.

## Methods

Baseline data in the present study were derived from the Norwegian study on outcomes research and quality improvement (RESQUA), a cross-sectional postal survey of HRQOL and the experiences of patients receiving hospital care [27]. All patients from surgical and internal medicine wards at 17 hospitals (4 teaching hospitals, 6 central hospitals, and 7 local hospitals) between October and December 1998 were sent a questionnaire 6 weeks after hospital discharge. No response within 4 weeks triggered one reminder. Patients younger than 16 years and those registered as dead at discharge were excluded from the study.

Participants with CHD discharged from internal medicine wards were selected for a follow-up postal survey approximately 2 years later, in October and November 2000. We used information on primary and secondary diagnoses from the patient-administration systems of the hospitals, and included patients with acute coronary syndrome (ICD-9 410.xx and 411.xx) and angina pectoris (ICD-9 413.xx). Patients with chronic heart failure (ICD-9 428.xx) as the primary diagnosis were classified as angina pectoris or acute coronary syndrome dependent on their secondary diagnoses.

## Measures

### Health-related quality of life

HRQOL was measured by the Norwegian version 1.2 of the Short-Form 36 (SF-36), a widely used generic health status measure that enables comparison with normative scores [28,29]. The scales and items of the SF-36 have satisfactory reliability, validity, and responsiveness, also in patients with CHD [3,10,30,31]. Single items of the SF-36 are transformed and aggregated into eight multi-item scales: Physical Functioning, Physical Role Limitations, Bodily Pain, General Health Perceptions, Vitality, Social Functioning, Emotional Role Limitations, and Mental Health. The resulting summated rating scales range from 0 to 100, with higher scores indicating better health.

To estimate the potential impact of CHD on HRQOL, we compared the SF-36 scores from the patients in our study with normative data from the Norwegian general population [32]. Norm scores for the eight multi-item scales were adjusted to reflect age and sex distributions similar to those of the patients in the present study. These adjusted norm data for the eight multi-item scales were used to calculate the standardized Physical (PCS) and Mental (MCS) Component Summary scores [33].

### Procedures, sociodemographics, and comorbidity

Invasive coronary procedures referred to diagnostic and therapeutic procedures, such as coronary angiography, which contributes to diagnosis of potential coronary artery disease, and when followed by angioplasty, it can contribute to a relief from chest-pain as well as improve the prognosis in high-risk patients. Procedure codes were derived from the patient-administration systems of the hospitals in 1998 (Classification of Operations; version 3, 1995). We defined invasive coronary procedures as a dichotomous variable, differentiating between patients with and without invasive coronary procedures during baseline hospitalization in 1998. The age and sex data were also derived from the administration systems in 1998. Information about the highest level of educational attainment was obtained from self-reported data in the 1998 postal survey. This variable was heavily positively skewed, and we therefore created two groups: (1) below and equal to, and (2) above the median value in our cohort. As a crude estimate of the degree of comorbidity for each patient, we used the total number of secondary diagnoses registered in the administration databases during baseline hospitalization in 1998.

### Statistical analyses

Changes in HRQOL were only assessed in patients who had valid scores on all multi-item scales both in 1998 and 2000. We used χ^2^-statistics or the *t*-test for independent samples to analyze the extent of selective attrition, and differences in the use of invasive coronary procedures across characteristics of respondents.

Temporal changes in HRQOL were analyzed with paired-samples *t*-tests. As a measure of the minimally important difference in intra-individual scores, we calculated the standardized response mean, a distribution-based approach that compares temporal change by the standard deviation of change [34]. Standardized response means of 0.2–0.5, 0.5–0.8, and > 0.80 are regarded as small, moderate, and large, respectively [35].

Additionally, we applied multivariate linear regression analyses to determine the association of invasive coronary procedures and sociodemographic factors with PCS and MCS scores 2 years after baseline hospitalization. By including baseline PCS and MCS scores in the regression model, the regression coefficients of invasive coronary procedures and sociodemographic factors indicate the one unit increase in 2-year PCS and MCS scores, provided that baseline scores are held constant.

All analyses were performed separately in patients with angina pectoris and acute coronary syndrome. We chose a 5% statistical significance level. The Regional Medical Research Ethics Committee, the Data Inspectorate, and the Norwegian Board of Health approved the study.

## Results

**Figure 1:**
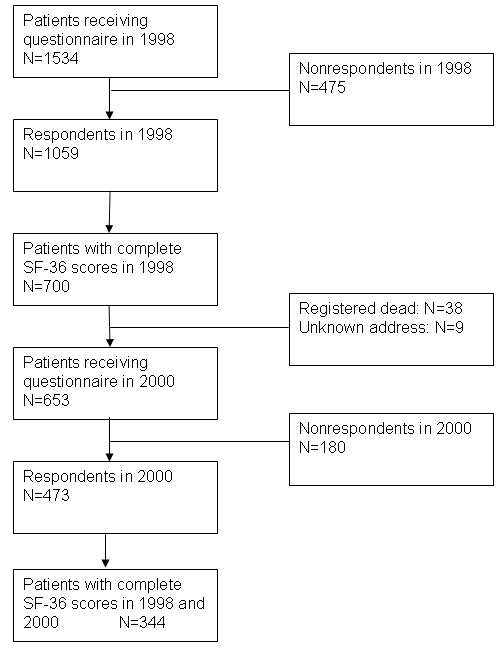
Flow chart describing attrition in the cohort of patients with coronary heart disease

A total of 1,534 patients with CHD were sent a questionnaire in 1998, and 1,059 (69%) responded. In 2000, 700 patients with valid HRQOL scores were sent a follow-up questionnaire (and, where necessary, one reminder), and 473 patients responded. After excluding 38 patients who had recently died and 9 patients with an unknown address, the adjusted response rate in the follow-up study was 72% (Figure 1).

A total of 254 patients with angina pectoris and 90 patients with acute coronary syndrome had valid MCS and PCS scores both in 1998 and 2000, of which 108 patients with angina pectoris and 41 patients with acute coronary syndrome underwent an invasive coronary procedure. The majority underwent catheterization (N = 79) or percutaneous coronary intervention (N = 45). Twenty-one patients underwent coronary bypass surgery and the remaining patients (N = 4) underwent other medical procedures related to the cardiovascular system. Compared to the original cohort of patients, patients with valid HRQOL scores both in 1998 and 2000, more often had undergone invasive coronary procedures, were male, younger, and had lower comorbidity (Table 1). Compared to the cohort with valid responses in 1998, attrition was associated with age, educational attainment and comorbidity. Angina pectoris patients who responded both in 1998 and in 2000 had higher PCS scores in 1998 compared to nonrespondents to the follow-up survey (mean HRQOL score 42 versus 39; *P *< 0.001). Among patients with valid HRQOL scores both in 1998 and 2000, women, elderly patients, and patients with higher comorbidity had fewer invasive coronary procedures during the baseline hospitalization (Table 2). Educational attainment was not associated with invasive coronary procedures.

**Table 1:** Baseline characteristics of respondents and non-respondents

	I. Total	II. Non-respondents at 6 weeks	III. Respondents at 6 weeks^a^	IV. Respondents at 6 weeks & 2 years^a^	Comparison Column IV and total nonresponse (*P*-value)	Comparison Column IV and nonresponse at 6 weeks (*P*-value)
	*N *= 1534	*N *= 475	*N *= 700	*N *= 344		
Age, mean (SD)	69 (12)	71 (12)	65 (11)	64 (10)	<0.001	0.001
Gender (% women)	34	44	29	27	0.001	0.2
> 10 years education (%)			44	49		0.014
> 1 diagnosis (%)	65	72	60	56	0.001	0.039
Emergency admission (%)	70	80	62	58	<0.001	0.07
Acute Coronary Syndrome (%)	31	32	28	26	0.047	0.4
Invasive Coronary Procedure (%)	33	22	40	43	<0.001	0.06
Teaching Hospital (%)	54	48	57	59		
Central Hospital (%)	26	27	25	22	0.042	0.123
Local Hospital (%)	20	25	18	19		

^a^Respondents to all multi-item scales of the SF-36

**Table 2:** Invasive coronary procedures (ICP) according to characteristics of baseline hospitalization and sociodemographic characteristics in patients with angina pectoris and acute coronary syndrome

	Angina pectoris		Acute coronary syndrome	
	No ICP	ICP	No ICP	ICP
Baseline characteristics	*N *= 146	*N *= 108	*N *= 49	*N *= 41
Sex				
Men (%)	70	77	73	76
Women (%)	30	23	27	24
Education				
≤ 10 years (%)	53	52	53	44
> 10 years (%)	47	48	47	56
Age (mean; (SD))	64 (11)	61 (9)	68 (9)	62 (10)
Type of hospital				
Teaching (%)	34	98	31	90
Central (%)	33	2	36	10
Local (%)	33	0	34	0
Length of hospital stay (mean (SD))	3.8 (3.1)	3.9 (3.7)	8.5 (4.8)	7.1 (7.3)
No. of diagnoses (mean (SD))	1.9 (1.1)	1.6 (0.9)	2.9 (1.2)	2.2 (1.0)

Six weeks after hospitalization, patients with angina pectoris and acute coronary syndrome had lower scores compared to the Norwegian norm data in all domains of HRQOL (Table 3 [see Additional file 1]). Patients without invasive coronary procedures exhibited the largest differences, particularly in domains referring to physical aspects of HRQOL: Physical Role Limitations, Emotional Role Limitations, and Bodily Pain; but also in General Health Perceptions. Two years after the baseline hospitalization in 1998, scores on all multi-item scales were still below the scores of the norm population.

Over the 2 years analyzed, Physical Role Limitations (*P *= 0.001) and Social Functioning (*P *= 0.003) improved in angina pectoris patients without invasive coronary procedures, corresponding to a small effect size (Table 3). Physical Functioning (*P *= 0.03), Physical Role Limitations (*P *< 0.001), and Bodily Pain (*P *= 0.03) improved in patients with angina pectoris undergoing invasive coronary procedures. The change in Physical Role Limitations corresponded to a moderate effect size. A significant deterioration was found in General Health Perceptions (*P *= 0.04).

In patients with acute coronary syndrome without invasive coronary procedures, Physical Role Limitations (*P *= 0.003), Social Functioning (*P *= 0.005), and Emotional Role Limitations (*P *= 0.009) significantly improved. Physical Role Limitations (*P *= 0.001) improved in patients with acute coronary syndrome undergoing invasive coronary procedures.

Patients with invasive coronary procedures showed a small improvement in PCS scores (*P *= 0.034 for angina pectoris and *P *= 0.015 for patients with acute coronary syndrome). MCS scores remained stable during the 2 years of follow-up; only patients with angina pectoris without an invasive coronary procedure experienced a small improvement in MCS scores (*P *= 0.007).

Multiple linear regression analyses revealed that, after taking baseline PCS scores into account, invasive coronary procedures and being younger, male, and more educated were significantly associated with higher PCS scores in 2000 in patients with angina pectoris (Table 4 [see Additional file 2]). For these patients, being older was significantly associated with higher MCS scores in 2000. In patients with acute coronary syndrome, PCS scores and MCS scores in 2000 were significantly associated only with baseline scores, and not with invasive coronary procedures or sociodemographic characteristics.

## Discussion

In the present study, most improvement was found in the physical components of HRQOL 2 years following invasive coronary procedures. These results support the findings of Krumholz et. al. [21] that the SF-36 scale for Physical Role Limitations was most responsive after elective coronary angioplasty. Furthermore, in patients with angina pectoris, PCS scores improved more among those who were male, younger, and more educated, independently of invasive coronary procedures. One explanation for this, as suggested by some previous studies, is related to differences in disease severity: women and patients from disadvantaged socioeconomic strata may have more extensive coronary disease at the onset of symptoms [12,13,36]. Additionally, undesirable events and adverse experiences might have stronger negative emotional consequences in this group [37], suggesting worse adaptation to the long-lasting physical limitations of CHD and a greater risk of recurrent events [36].

When adjusting for baseline scores, invasive coronary procedures and sociodemographic characteristics did not explain any additional variation in PCS and MCS scores 2 years after hospitalization in patients with acute coronary syndrome. This may be due to the relatively small sample size. An alternative explanation is that patients with acute coronary syndrome exhibited higher comorbidity that could limit the effect of invasive coronary procedures on HRQOL, and accordingly, the sensitivity of the SF-36 in detecting differences [20].

Our results demonstrated that invasive coronary procedures and sociodemographic characteristics were weakly associated with MCS scores and indicated small deviations from the population norm, which corresponds to previous findings in patients with CHD [6,38]. This may be attributable to health care having less impact on mental health than on physical health. An alternative explanation refers to the construction of the SF-36 MCS and PCS measures. The scores of these component scales are calculated using all eight multi-item scales with factor score coefficients derived from factor analysis with orthogonal rotation, thereby defining that PCS and MCS are uncorrelated. Mean scores on the multi-item scales that are below the population mean will contribute to component scores opposite to the direction defined by the factor score coefficient [39]. In our study, the low scores of Physical Role Limitations contributed negatively to PCS and positively to MCS. Hence, MCS scores were probably inflated by poor physical health. The RAND-36 has been suggested as an alternative method for computing PCS and MCS scores that avoids the orthogonal approach of the SF-36 [40,41].

Other factors may have influenced our results, for example selective attrition. In our study, the respondents to both surveys represent a survivor cohort, and hence attrition may have reduced the temporal changes in SF-36 scores and possibly lead to underestimation of the associations with invasive coronary procedures and sociodemographic factors. Moreover, the use of self-administered and postal questionnaires may have contributed to missing SF-36 items, especially in elderly subjects who are associated with a higher frequency of missing values for items used to score physical and emotional role functioning [24]. The appropriateness of the SF-36 for use in elderly populations with expected low response rates, reduced cognitive functioning, and shifts in conceptualizations of subjective health, has been discussed previously [32]. Consequently, caution should be exercised when employing norms among people aged 70 years and older.

Another limitation of our study is the lack of HRQOL data before the baseline hospitalization in 1998, which prevented us from assessing the full impact of invasive coronary procedures on subsequent HRQOL and its association with sociodemographic characteristics. We also did not examine the influence of use of medical services after the baseline hospitalization. Finally, coronary patients and invasive procedures were defined by registry data from the patient-administration systems of hospitals, which might be inaccurate and mask some of the underlying clinical differences that could influence the HRQOL results.

Our findings indicated that patients with angina pectoris who were younger, male, and more educated were most likely to increase their HRQOL following invasive coronary procedures. In patients hospitalized for acute coronary syndrome, temporal change in HRQOL was not associated with invasive coronary procedures and sociodemographic characteristics, possibly due to higher comorbidity. In a usual care setting the occurrence of invasive coronary procedures varies with sociodemographic characteristics [42,43]. The association of both sociodemographic variables and invasive coronary procedures with HRQOL outcomes makes it imperative to take these into account when comparing and interpreting change scores to reduce the risk of spurious findings.

## Authors' contributions

MV carried out the follow-up study, analyzed the data, and drafted the manuscript. KIP performed the baseline survey. AR participated in the design of the study. KS participated in the design and coordination of the study. All authors read and approved the final manuscript.

## Supplementary Material

Additional File 1 Table 3: SF-36 multi-item and summary scales 6 weeks and 2 years after baseline hospitalization in patients with angina pectoris and acute coronary syndrome according to invasive coronary procedures (ICP). Norwegian norm data and 2-year change scores Click here for file

Additional File 2 Table 4 Predictors of Physical Component Summary (PCS) and Mental Component Summary (MCS) scores 2 years following the baseline hospitalization in patients with angina pectoris and acute coronary syndrome. Multivariate linear regression analysis; unstandardized regression coefficients (*B*) and 95% Confidence Intervals (CI) Click here for file
